# Deep Learning-Based Methods for Prostate Segmentation in Magnetic Resonance Imaging

**DOI:** 10.3390/app11020782

**Published:** 2021-01-15

**Authors:** Albert Comelli, Navdeep Dahiya, Alessandro Stefano, Federica Vernuccio, Marzia Portoghese, Giuseppe Cutaia, Alberto Bruno, Giuseppe Salvaggio, Anthony Yezzi

**Affiliations:** 1 Ri.MED Foundation, Via Bandiera, 11, 90133 Palermo, Italy;; 2 Institute of Molecular Bioimaging and Physiology, National Research Council (IBFM-CNR), 90015 Cefalù, Italy; 3 Department of Electrical and Computer Engineering, Georgia Institute of Technology, Atlanta, GA 30332, USA;; 4 Dipartimento di Biomedicina, Neuroscienze e Diagnostica avanzata (BIND), University of Palermo, 90127 Palermo, Italy;

**Keywords:** deep learning, segmentation, prostate, MRI, ENet, UNet, ERFNet, radiomics

## Abstract

Magnetic Resonance Imaging-based prostate segmentation is an essential task for adaptive radiotherapy and for radiomics studies whose purpose is to identify associations between imaging features and patient outcomes. Because manual delineation is a time-consuming task, we present three deep-learning (DL) approaches, namely UNet, efficient neural network (ENet), and efficient residual factorized convNet (ERFNet), whose aim is to tackle the fully-automated, real-time, and 3D delineation process of the prostate gland on T2-weighted MRI. While UNet is used in many biomedical image delineation applications, ENet and ERFNet are mainly applied in self-driving cars to compensate for limited hardware availability while still achieving accurate segmentation. We apply these models to a limited set of 85 manual prostate segmentations using the k-fold validation strategy and the Tversky loss function and we compare their results. We find that ENet and UNet are more accurate than ERFNet, with ENet much faster than UNet. Specifically, ENet obtains a dice similarity coefficient of 90.89% and a segmentation time of about 6 s using central processing unit (CPU) hardware to simulate real clinical conditions where graphics processing unit (GPU) is not always available. In conclusion, ENet could be efficiently applied for prostate delineation even in small image training datasets with potential benefit for patient management personalization.

## 1. Introduction

In the biomedical imaging field, target delineation is routinely used as the first step in any automatized disease diagnosis system (i.e., radiotherapy system) and, in the last few years, in radiomics studies [1,2] to obtain a multitude of quantitative parameters from biomedical images [3,4]. These parameters are then used as imaging biomarkers to identify any possible associations with patient outcome. The first task of a radiomics analysis is the automatic and user-independent target (e.g., tumor or organ) delineation to avoid any distortion during the feature extraction process [5]. Manual segmentation might seem like the simplest solution to obtain target boundaries, but it is a time-consuming and user-dependent process that affects the radiomics signature [6]. For this reason, an automatic and operator-independent target delineation method is mandatory. Nevertheless, the segmentation process remains a challenging field of research. Over the years many different types of segmentation techniques have been developed, for example, [7–9]. Some of the previous techniques include thresholding [10], k-means clustering [11], watersheds [12], followed by more advanced algorithms such as active contour methods [8,13], graph cuts [14], random walks [15], conditional and Markov random fields [16] to name a few. In recent years, particularly the last decade, the field of Machine Learning (ML) and Deep Learning (DL) has seen exponential growth and has produced models that have shown remarkable performance across many benchmark datasets and many different problem domains [17,18]. In general, an artificial intelligence method learns from examples and makes predictions without prior specific programming [19]. In the case of DL, these models implement networked structures to mimic the human brain transforming imaging data in feature vectors. Briefly, between the input and output, a variable number of hidden layers is implemented and the various nodes are connected to others with different weights.

The initial development of DL models was towards image classification problems, followed by object detection and finally, image segmentation, which is seen as a pixel level classification problem where each pixel is classified with one of many possible label classes. For example, in tumor segmentation, every voxel can be classified as either belonging to the class label of the object of interest (target) or the background. Since it is a very common task across many different problem domains, hundreds of different DL based models have been presented for the delineation task over the past several years: fully convolutional [20], encoder-decoder [21], multi-scale and pyramid [22–24], attention [25], recurrent neural [26], generative and adversarial training [27,28] based networks. Even during the current pandemic, DL networks have been widely used to help clinicians diagnose COVID-19 [29,30]. It is beyond the scope of this paper to discuss and describe all these different types of models. Interested readers are directed to recent comprehensive reviews [31,32] of DL based methods/models for image segmentation.

In this study, we deal with the issue of prostate region delineation on magnetic resonance imaging (MRI) studies. Prostatic volume extraction helps in the planning of biopsies, surgeries, focal ablative, radiation, and minimally invasive (e.g., intensity focused ultrasound [33]) treatments. In addition, benign prostatic hyperplasia, also called prostate enlargement, is one of the most common conditions affecting men [34]. A correlation between prostatic volume, and the incidence of prostate cancer, where early tumor identification is crucial to reduce mortality, has been shown in [35]. Since only part of prostate cancer is clinically significant, risk stratification is mandatory to avoid over-diagnosis and over-treatment [36,37]. For this reason, radiomics in MRI has acquired a crucial role in the risk stratification process [19,36]. MRI allows calculating prostatic volume considering the prostate as an ellipsoid. Unfortunately, the shape of the prostate varies and the determination of its volume based on the ellipsoid formula is often incorrect [38]. The presence of prostate cancer may alter the prostate volume as reported, for example, in the study of [39]: the authors reported that shape differences in the prostate gland were consistently observed between patients with or without prostate cancer maybe as the result of cancer localized in the peripheral zone. For this reason, the manual delineation is more accurate than the previously described method but takes time, requires experience, and is highly operator-dependent as noted above. Consequently, several automatic algorithms have been proposed, for example, [40–42]. Due to the lack of large amounts of labeled data for the training process, DL is still far from a widespread application in the biomedical environment. So, there is a need to develop DL networks to obtain accurate delineations with fewer training examples. Then, we explore the efficacy of Efficient Neural Network (ENet) [43] and Efficient Residual Factorized ConvNet (ERFNet) [44] that are mainly applied in self-driving cars to compensate for limited hardware availability while still achieving accurate segmentation, and UNet that is used in many biomedical image delineation applications [45]. Using a limited set of 85 manual prostate segmentation training data, we show that ENet model can be used to obtain accurate, fast and clinically acceptable prostate segmentations.

## 2. Materials and Methods

### 2.1. Experimental Setup

To test DL based methods for prostate segmentation, we used prostate studies of patients who underwent MRI examinations using the Achieva scanner (Philips Healthcare, Best, The Netherlands) with a pelvic phased-array coil (8 channel HD Torso XL). Specifically, from September 2019 to May 2020, 202 consecutive patients were referred to our Radiology Department to perform a prostate MRI examination. We excluded patients from the study for (a) incomplete MRI examination due to intolerance, discomfort, or claustrophobia (n = 11); (b) patients with radical prostatectomy (n = 18), subjected to transurethral resection of the prostate (TURP) (n = 20), or radiotherapy (n = 17); (c) lack of median lobe enlargement defined as intra-vesical prostatic protrusion characterized by overgrowth of the prostatic median lobe into the bladder for at least 1 cm (n = 51). So, our final study population consisted of 85 patients (age range 43–75 years, mean age 59 ± 8.4 years) with median lobe enlargement. By reviewing radiological reports, no pathological MRI findings were found in 35 patients (except for median lobe enlargement), while 50 prostate lesions (42 in peripheral zone and 8 in transitional zone) suspected for prostate cancer classified using the Prostate imaging reporting and data system (PI-RADS) 2.1 [46] were found: 18 PI-RADS 3 score, 28 PI-RADS 4 score, and 4 PI-RADS 5 score lesions with size ranged between 0.6 and 1.9 cm (mean 1.052 ± 0.28). In addition, in our study population, by evaluating capsular involvement, 18 patients had capsular abutment and 3 patients had capsular irregularity. It means that the presence of suspected prostate cancer lesions, in our study population, can at least distend the gland boundaries. Consequently, the determination of prostate volume using the above mentioned ellipsoid formula [38] is not suitable, while manual and automatic segmentations are not (or less) affected by this issue.

In this study, axial T2-weighted images with parameters shown in Table 1 were used. However, due to MRI protocol routine update during the study time, datasets had different resolution (2 studies with a matrix resolution of 720 × 720; 45 studies with a matrix resolution of 672 × 672; 23 studies with a matrix resolution of 576 × 576; 15 studies with a matrix resolution of 320 × 320). Consequently, the datasets had different resolutions and sizes. Since DL networks require inputs of the same size for the training process, MRI images were resampled to the isotropic voxel size of 1 × 1 × 1 mm^3^ with a matrix resolution of 512 × 512 (matrix resolution in the middle between 720 and 320) using linear interpolation. A set of trained clinical experts (FV, MP, GC, and GS authors) hand segmented the prostate region. The simultaneous ground truth estimation STAPLE tool [47] was used to combine the different segmentations from the clinical experts in a consolidated reference. Finally, manual delineations were resampled using nearest neighbor interpolation and converted to masks with 0 for the background and 1 for the prostate area.

### 2.2. Deep Learning Models

Three different deep learning models including UNet [45], ENet [43], and ERFNet [44] were investigated to account for accurate prostate segmentation, fast training time, low hardware requirements for inference, and low training data requirements. Specifically, UNet was modified to improve segmentation accuracy, as reported in [48,49]. Briefly, (i) 3 × 3 convolutions were replaced by 5 × 5 convolutions, (ii) zero padding was used to ensure that the size of the output feature maps was the same as the input size, and (iii) an input size of 512 × 512 with 32 filters was used on the first contraction path layer, with doubling of feature maps after each max pool and stopping at 256 feature maps and 2D size of 64 × 64.

Concerning ENet and ERFNet (see Table 1 in [43] and Table 2 in [44] for the description of their architecture), they were mainly applied in self-driving cars to compensate for limited hardware availability maintaining high accuracy and successfully used in two biomedical segmentation issues [48,49], that is, in the segmentation of high resolution computed tomography (HRCT) images characterized by a slice thickness much lower than that of the T2 weighted images of the prostate studies. This means that the number of slices of each patients’ study was much greater than in this study.

### 2.3. Training Methodology

Due to a limited amount of data, the k-fold cross-validation strategy was applied by randomly dividing the dataset into k sub-datasets of equal size (17 patients, k = 5). For each network, we trained k models by combining k-1 folds into the training set and keeping the remaining fold as a holdout test set. Despite the fact that 2D models were considered, slices from the same study were never used for both training and testing purposes. So, there was no cross-contamination between training and test sets.

Moreover, the data augmentation technique was applied in six different modalities to increase the statistic. Additionally, data standardization and normalization were adopted to prevent the weights from becoming too large, to make the model converge faster, and to avoid numerical instability. Regarding loss function, prostate segmentation suffers from the imbalanced data problem because there are very few examples of the positive class compared to the background or negative class. In terms of image segmentation, the target (i.e., the prostatic region) is small compared to the background, which may be composed of many different organs or types of tissue exhibiting a wide range of intensity values. Some slices may have a very small target area compared to the background. This makes it hard for the DL to learn a reliable feature representation of the foreground class. In such cases, the networks tend to simply predict most voxels as belonging to the background class. To deal with this problem, various loss functions have been proposed over the years. These loss functions typically aim to solve the class imbalance problem by providing a larger weight to foreground voxels. This translates to a higher penalty in the loss function for foreground voxels that are misclassified by the network leading to the network being able to learn the foreground object representation more effectively. One such loss function which the authors of this paper have experimentally determined to be better suited for the biomedical image delineation process is the Tversky loss function [50]. Specifically, the Dice similarity coefficient (DSC) between *P* and *G* is defined as:

(1) $$\text{DSC}=\frac{2\left|P\cap G\right|}{\left|P\right|+\left|G\right|}$$

where *P* and *G* are the predicted and ground truth labels. DSC measures the overlap between *P* and *G* and is used as a loss function in many DL approaches. Nevertheless, DSC is the harmonic mean of false positives and false negatives and weighs both equally. To modify their weights, the Tversky index [51] was proposed as:

(2) $$S(P,G;\alpha \;\beta )=\frac{\left|P\cap G\right|}{\left|P\cap G\right|+\alpha \left|P\setminus G\right|+\beta \left|G\setminus P\right|}$$

*α* and *β* control the penalty magnitude of false positives and false negatives. Using this index, the Tversky loss [50] is defined as:

(3) $$T(\alpha \;\beta )=\frac{\sum_{i=1}^{N}\;p_{0i}\;g_{0i}}{\sum_{i=1}^{N}\;p_{0i}\;g_{0i}+\alpha \sum_{i=1}^{N}\;p_{0i}\;g_{1i}+\beta \sum_{i=1}^{N}\;p_{0i}\;g_{0i}}$$

**Figure 1.**
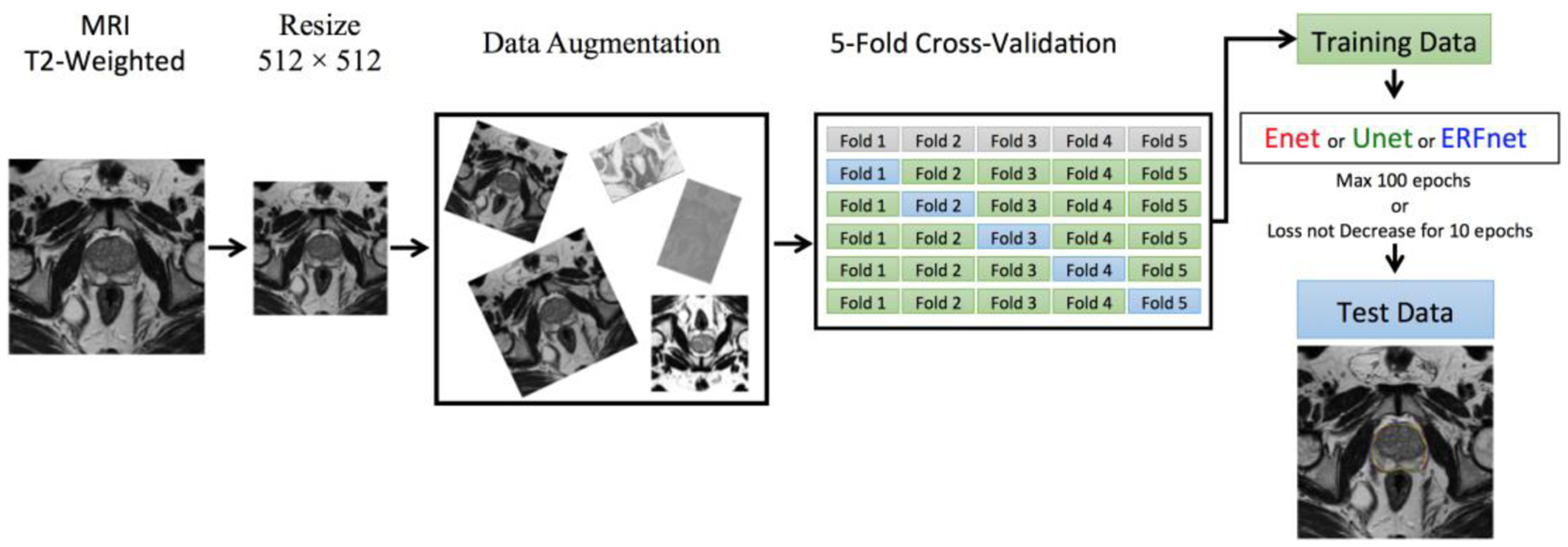
Workflow of the proposed segmentation method.

Additional information about the study design is shown in Figure 1.

**Figure 2.**
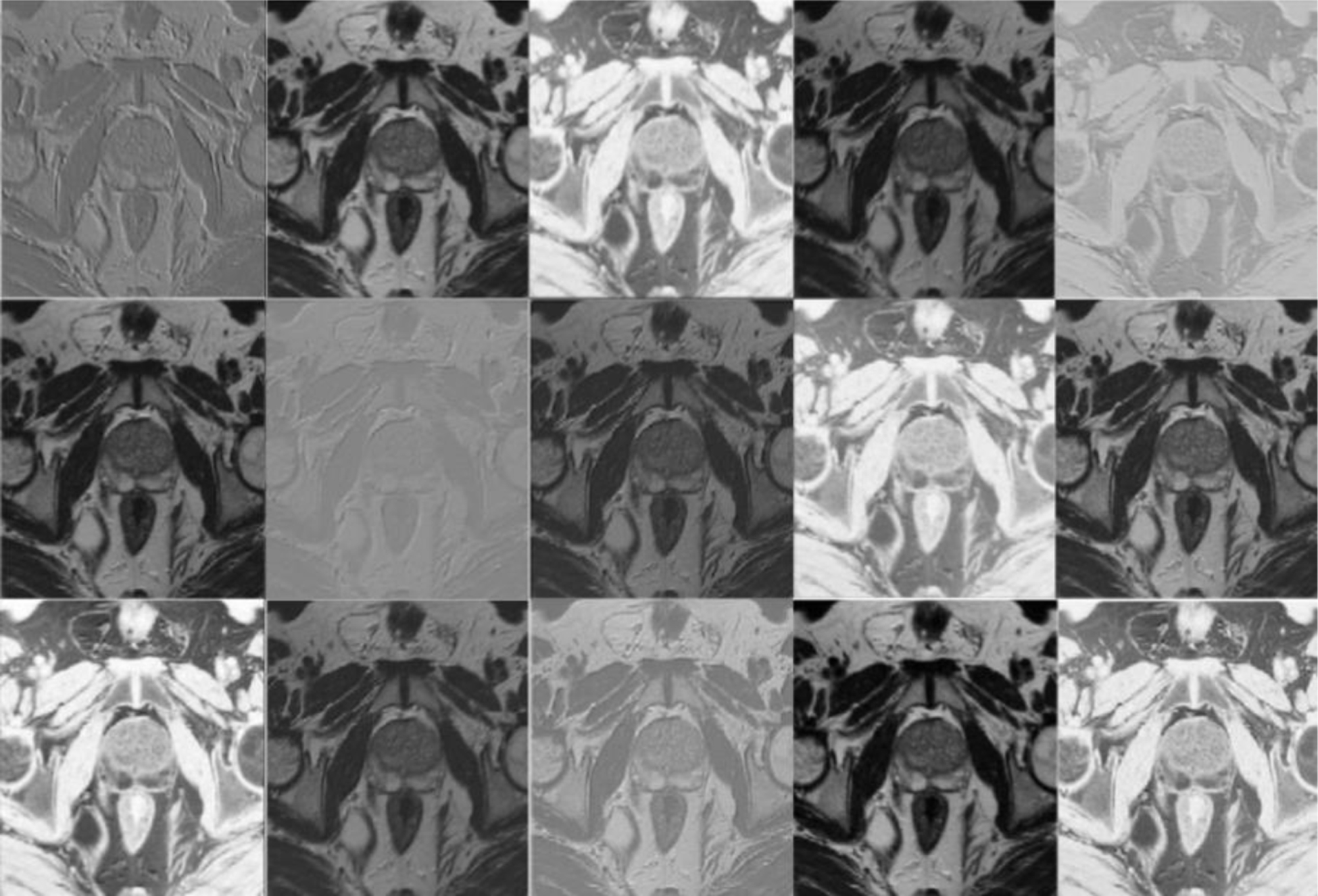
Feature maps (None, 256, 256, 15) extracted from the first hidden layer in the ENet Model for Patient #7 slice #20.

Starting from 16 patients, the best learning rates for each network were determined experimentally. We used a learning rate of 0.0001 for ENet, 0.00001 for ERFNet, and UNet with Adam optimizer [52]. A batch size of eight slices, *α* and *β* of 0.3 and 0.7, respectively for the Tversky loss function, were identified. All the models were allowed to train for a maximum of 100 epochs with an automatic stopping criteria of ending training when the loss did not decrease for 10 epochs continuously. The GEFORCE RTX 2080 Ti with 11GB of RAM (NVIDIA) was used to train DL models and run inference. Table 2 and Figure 2 show the feature representation learned from the first hidden layer in the ENet model.

## 3. Results

Sensitivity, positive predictive value (PPV), DSC, volume overlap error (VOE), and volumetric difference (VD) were used for performance evaluation:

(4) $$\text{Sensitivity }=\frac{TP}{TN+FN}$$

(5) $$\text{PPV}=\frac{TP}{TP+FN}$$

(6) $$\text{DSC}=\frac{2TP}{2TP+FP+FN}$$

(7) $$\text{VOE}=1-\frac{TP}{TP+FP+FN}$$

(8) $$\text{DSC}=\frac{2TP}{2TP+FP+FN}DSC=\frac{2\left|P\cap G\right|}{\left|P\right|+\left|G\right|}$$

Table 3 shows the performance obtained using ENet, UNet, and ERFNet methods. In particular, ENet showed a mean DSC of 90.89 ± 3.87%, UNet of 90.14 ± 4.69%, and ERFNet of 87.18 ± 6.44%.

Analysis of variance (ANOVA) based on DSC was calculated to test statistical differences (a *p*-value < 0.05 indicates a significant difference) between methods considering all patients (n = 85). Table 4 shows how though ENet and UNet minimized the difference between manual and automated segmentation.

Despite the fact that they were statistically identical, they were computationally different. ENet is much faster than UNet. Specifically, Table 5 shows the comparison of computational complexity and performance of the three models. As both ENet and ERFNet were developed for real-time applications, these are relatively smaller and faster than UNet. As shown in the table, the ENet model has an order of magnitude with fewer parameters than both ERFNet, and UNet while ERFNet has less than half the number of parameters compared to UNet. Consequently, the size of trained ENet is only 6 MB compared to 65 MB for the UNet model. To estimate the delineation time, we considered one of the trained models during the k-fold strategy for all three architectures and then computed the average. Using a fairly advanced GPU device (GEFORCE RTX 2080 Ti, 11 GB VRAM, 4352 CUDA Cores, NVIDIA), it takes only 1 s for ENet and about 1.5 s for UNet to generate segmentation on a 3D dataset (average 40 slices of 512 × 512). However, when GPU hardware is not available then computation needs to be done on the CPU. In such a scenario, the size of a model can play a big role. On an AMD Ryzen 2950x processor, ENet only takes about 6 s while UNet takes about 40 s to delineate a study. Soon, this computational advantage of ENet may make it possible to use this model to segment cases on simple hardware like IPads or smartphones for faster clinical workflow. Finally, only the ENet model makes use of batch normalization layers, which have some parameters which are not trained, that is, gradients are not back-propagated during the training process.

**Figure 3.**
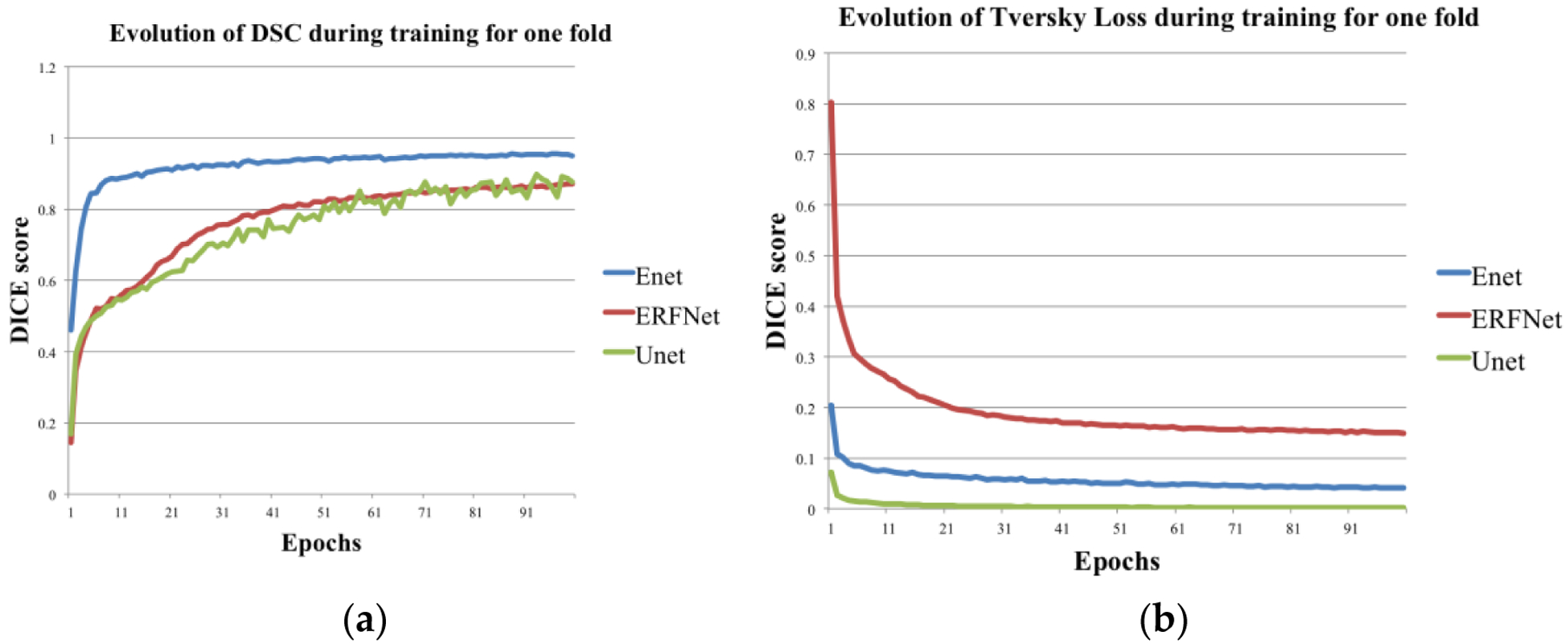
(**a**) Training DSC and (**b**) loss function Tversky loss plots for each of the three models for one fold.

**Figure 4.**
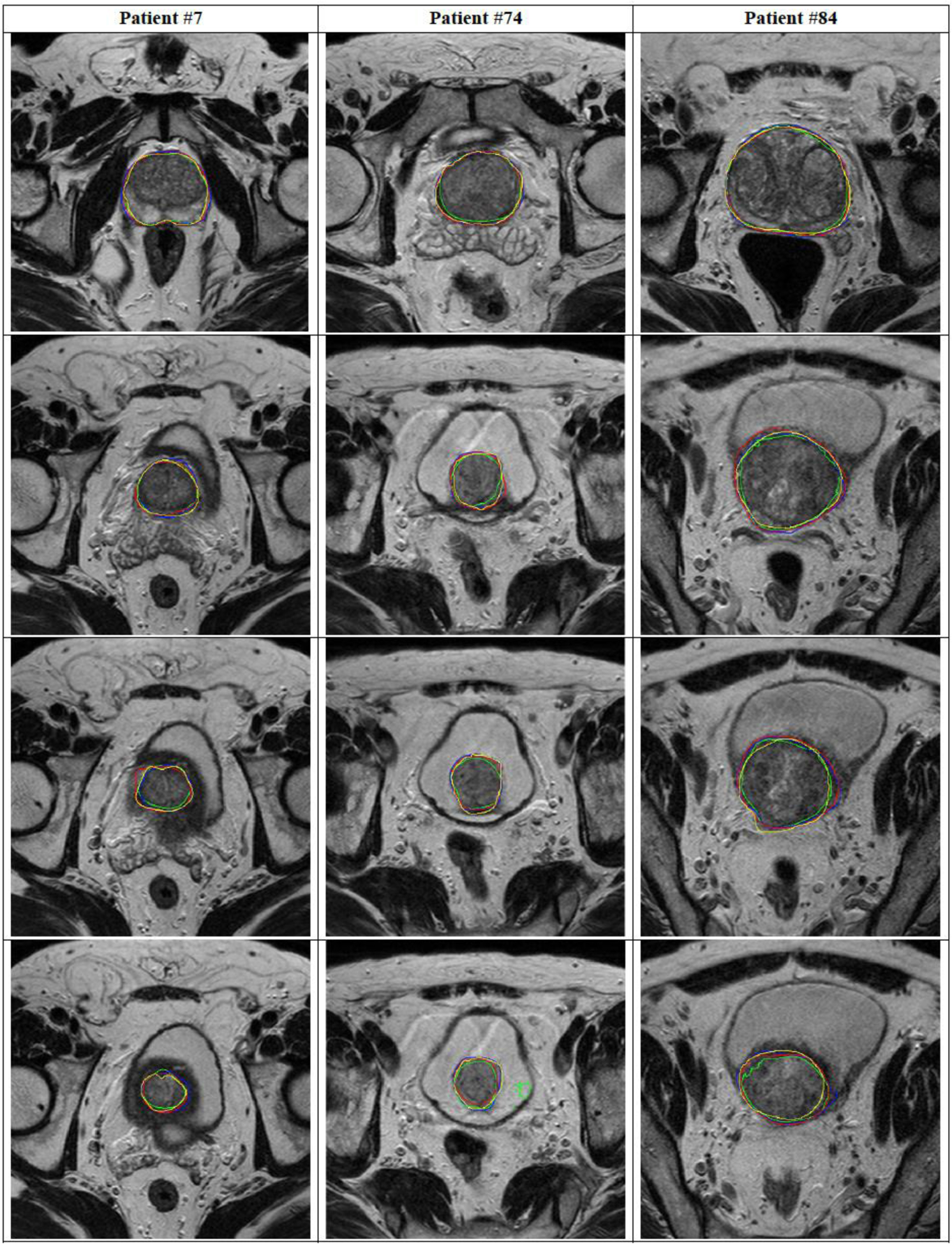
Comparison of segmentation performance for the three Net architectures in #7, 74, and #84 patients (four different slices for each patient). The manual segmentation (yellow), ENet (red), ERFNet (blue), and U-Net (green) are superimposed.

**Figure 5.**
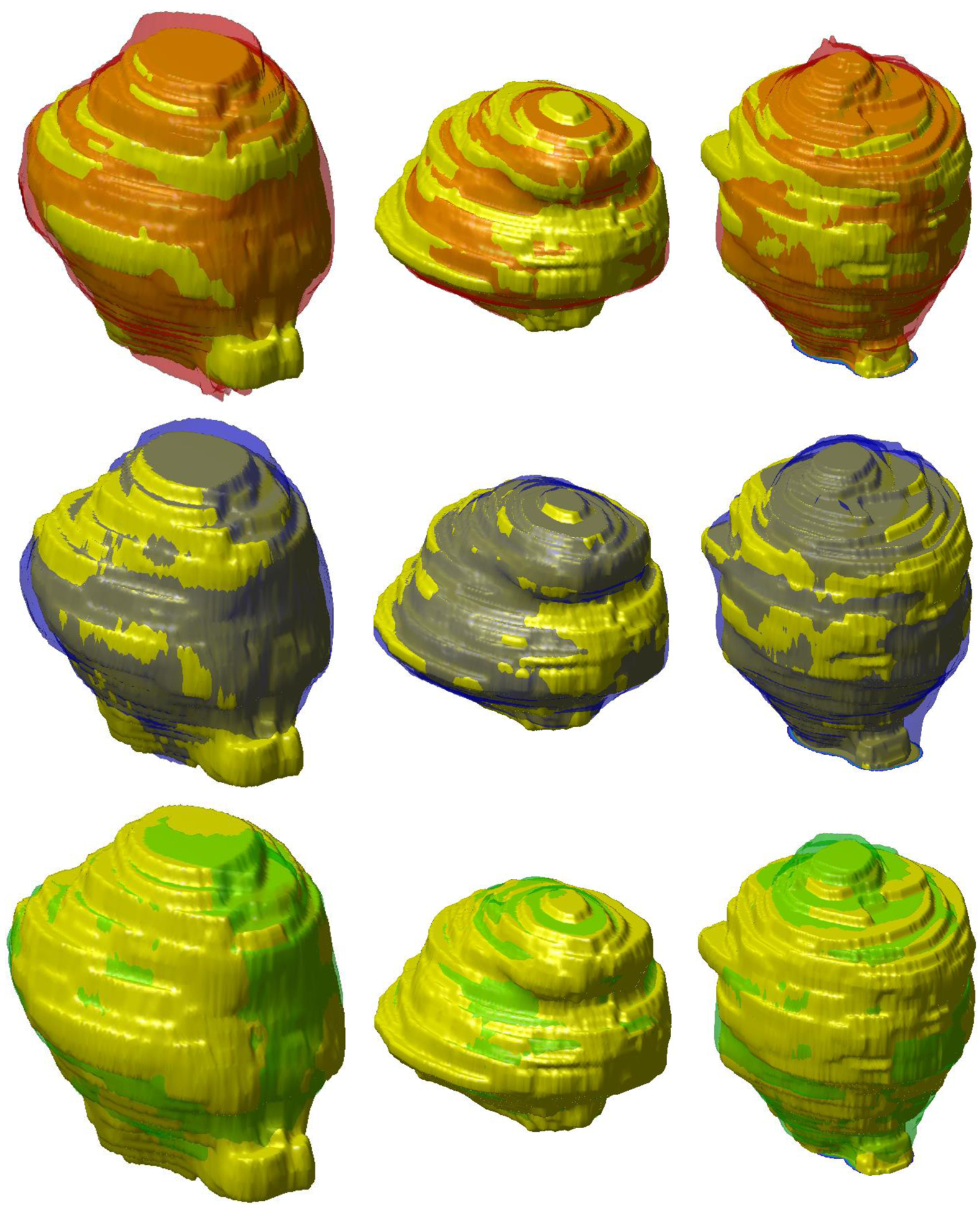
Comparison of 3D segmentation of prostate (patients #7, #74, and #84) for each column using the three Net architectures. The manual segmentation (yellow), ENet (red), ERFNet (blue), and U-Net (green) are superimposed.

In Figure 3, we plot the training DSC and Tversky loss function for each DL network for one fold. DSC and Tversky loss plots indicate that the ENet model converges much faster than both ERFNet and UNet. ENet model reaches a DSC = 0.85 in less than 15 epochs. Consequently, it is much faster to train a new ENet model compared to the other two if more training data become available in the future. Another noticeable feature is that the UNet training loss is much less compared to ENet and ERFNet, indicating the presence of overfitting. It can be concluded that even though ENet and UNet models are not statistically different, it may be advantageous to prefer ENet over UNet. Finally, 2D and 3D segmentation examples of three patient studies are shown in Figures 4 and 5, respectively.

## 4. Discussion

In this paper, we investigate the prostatic region segmentation in MRI studies using three different DL networks (namely UNet, ENet, and ERFNet). The aim is to reduce patient mortality being only a part of prostate cancer that is clinically significant. An accurate and operator-independent segmentation process is needed to obtain a relevant texture-based prediction model. So, the aim of this work was not only just to test the segmentation results of the proposed models, but to evaluate if these models can yield a practical benefit in obtaining accurate and reproducible results. The inclusion of DL models in radiomics analyses will be reserved for a forthcoming paper. The first model considered in this study was UNet, which has been adopted in several image delineation processes [45]. ENet [43], and ERFNet [44] have been implemented for the segmentation process in self-driving cars, and successfully used in lung and aorta segmentation tasks [48,49]. Specifically, they were used for the segmentation of HRCT images characterized by a very high number of slices for each study (about 600 and 450 slices for the lung and aorta studies, respectively). Authors used 32 patients’ studies for the parenchyma extraction process [49], and 72 studies for the aorta segmentation process [48]. In this study, only 85 studies were used considering that each patients’ image dataset consists of about 40 slices. In addition, to our knowledge, these DL models have never been applied to prostate segmentation before.

In general, a DL approach requires a multitude of labeled data for training and validation purposes. For this reason, DL models are not widely used in clinical practice. As already reported in the Introduction section, there is a need to develop DL networks capable of obtaining accurate segmentations with few training examples. This issue is addressed in some studies, that is, the one-shot learning approach [53], which eliminates the need for iterative sample selection and annotation and the contrastive learning method [29] for the automated diagnosis of COVID-19 with few samples for training. In our study, we applied all three DL models to a small dataset of 85 studies provided with manual prostate segmentation adopting (i) a data augmentation strategy to reduce overfitting, (ii) a data standardization and normalization to prevent too large weights, to make the model converge faster, and to avoid numerical instability, (iii) the five-fold cross-validation strategy to obtain good results despite the few training examples, and (iv) the Tversky loss function [50] to avoid to predict most voxels as belonging to the background class. In the last case, starting from the consideration that DL methods suffer from the imbalanced data problem because the target (i.e., the prostate) is very small compared to the background, we provided a larger weight to target voxels to learn the foreground object representation more effectively. Finally, we compared the obtained performances showing that accurate and clinically acceptable prostate segmentations with few training examples were obtained using indifferently the three DL models (DSC > 87%).

Specifically, results showed that ENet and UNet had better performance in minimizing the difference between automated and manual segmentations than ERFNet. Substantially, ENet and UNet were statistically identical but computationally different; ENet was much faster than UNet (see Figure 3). Also, the training Tversky loss of the UNet was much less compared to ENet. For these reasons, though UNet and ENet were not statistically different, ENet seems to be the best solution. This could justify the time required to include DL networks in radiomics analyses by removing the user-dependence and achieving accurate prostate segmentations (DSC = 90.89%) using a few training examples. In this way, our model can be used to improve prognosis evaluation and prediction of patient outcomes, allowing the personalization of patient management. However, the results presented in this study derive from the performance of DL networks on proprietary imaging datasets; for routine clinical application, it should be mandatory to test and validate the proposed methods in multicenter studies and/or on a large set of publicly released representative training data, such as PROMISE12 [42]. Moreover, in the present study, we test DL networks for the whole prostate gland segmentation, with ENet demonstrating the best performance; however, a main clinical goal is the segmentation of related prostatic structures or substructures such as the prostatic zones (transition, central and peripheral), neurovascular bundles or seminal vesicles. The performance of DL networks, especially ENet, on this topic should play an essential role in many medical imaging and image analysis tasks such as cancer detection, patient management, and treatment planning including surgical planning, and should be analyzed in future works. Automatic segmentation of the whole prostate gland and prostatic zone (transition, central and peripheral) without inter-user variability will lead, in the future, to a correct localization of prostate cancer. This result will increase the reliability of computer-aided design (CAD) algorithms which will help automatically create PI-RADS zone maps to reduce inter-user variability among clinicians when interpreting prostate MRI images. In this scenario, radiomics analysis should be performed automatically providing information that can lead clinicians on the management of patients with prostate cancer.

## 5. Conclusions

Our study demonstrates that faster and less computationally expensive DL networks can perform accurate prostate delineation and could facilitate the adoption of novel imaging parameters, through radiomics analyses, for prostatic oncologic diseases. Specifically, we assessed the performance of three DL networks using data augmentation, standardization, and normalization, and the five-fold cross-validation strategies, and the Tversky loss function in a small dataset of 85 studies. All DL networks achieved accurate prostate segmentations with a DSC > 87%. Nevertheless, differences related to training time and data requirements were highlighted. ENet and ERFNet, developed for self-driving cars, were much faster than UNet. In addition, ENet had better performance (DSC = 90.89%) than ERFNet (DSC = 87.18%). Future studies with more patients could improve the results.

## Figures and Tables

**Table 1.** Parameter of MRI protocol.

Parameter	Repetition Time (ms)	Echo Time (ms)	Flip Angle (Degrees)	Signal Averages	Signal-to-Noise Ratio
T2w TSE	3091	100	90	3	1

**Table 2.** The model parameters and shape output after the first hidden layer in the ENet model for a given provided input image (Patient #7 slice #20).

Layer (Type)	Output Shape	Parameters Number
input_l (InputLayer)	(None, 512,512,1)	0
conv2d_l (Conv2D)	(None, 256, 256,15)	150

**Table 3.** Performance segmentation using the ENet, UNet, and ERFNet methods.

	Sensitivity	PPV	DSC	VOE	VD
ENet					
Mean	93.06%	89.25%	90.89%	16.50%	4.53%
±std	6.37%	3.94%	3.87%	5.86%	9.43%
±CI (95%)	1.36%	0.84%	0.82%	1.24%	2.00%
UNet					
Mean	88.89%	91.89%	90.14%	17.66%	3.16%
±std	7.61%	3.31%	4.69%	6.91%	9.36%
±CI (95%)	1.62%	0.70%	1.00%	1.47%	1.99%
ERFNet					
Mean	89.93%	85.44%	87.18%	22.18%	5.70%
±std	10.92%	5.43%	6.44%	9.61%	14.72%
±CI (95%)	2.32%	1.16%	1.37%	2.04%	3.13%

**Table 4.** ANOVA on the DSC showed statistical differences between segmentation methods.

ANOVA	F Value	F Critic Value	*p*-Value
ENet vs. ERFNet	20.70407668	3.897407169	0.000010236
ERFNet vs. UNet	11.69135829	3.897407169	0.000788084
ENet vs. UNet	1.301554482	3.897407169	0.255553164

**Table 5.** Computational complexity of the three models.

Model Name	Number of Parameters		Size on Disk	Inference Times/Dataset	
	Trainable	Non-Trainable		CPU	GPU
ENet	362,992	8352	5.8 MB	6.17 s	1.07 s
ERFNet	2,056,440	0	25.3 MB	8.59 s	1.03 s
UNet	5,403,874	0	65.0 MB	42.02 s	1.57 s

## Featured Application:

The study demonstrates that high-speed deep learning networks could perform accurate prostate delineation facilitating the adoption of novel imaging parameters, through radiomics analyses, for prostatic oncologic diseases.
